# Prussian blue nanotechnology in the treatment of spinal cord injury: application and challenges

**DOI:** 10.3389/fbioe.2024.1474711

**Published:** 2024-09-11

**Authors:** XiaoPeng Gu, SongOu Zhang, WeiHu Ma

**Affiliations:** ^1^ Department of Clinical Medicine, Health Science Center, Ningbo University, Ningbo, Zhejiang, China; ^2^ Department of Orthopedics, NingBo NO.6 Hospital, Ningbo, Zhejiang, China; ^3^ Department of Orthopedics, Zhoushan Guhechuan Hospital, Zhoushan, Zhejiang, China; ^4^ Zhoushan Institute of Orthopedics and Traumatology, Zhoushan, Zhejiang, China

**Keywords:** spinal cord injury, Prussian blue nanotechnology, treatment, inflammation, oxidative stress

## Abstract

Spinal cord injury (SCI) is a serious neurological condition that currently lacks effective treatments, placing a heavy burden on both patients and society. Prussian blue nanoparticles exhibit great potential for treating spinal cord injuries due to their excellent physicochemical properties and biocompatibility. These nanoparticles have strong anti-inflammatory and antioxidant capabilities, effectively scavenge free radicals, and reduce oxidative stress damage to cells. Prussian blue nanotechnology shows broad application potential in drug delivery, bioimaging, cancer therapy, anti-inflammatory and oxidative stress treatment, and biosensors. This article reviewed the potential applications of Prussian blue nanotechnology in treating spinal cord injuries, explored the challenges and solutions associated with its application, and discussed the future prospects of this technology in SCI treatment.

## 1 Introduction

Spinal cord injury (SCI) is a severe neurological condition typically caused by trauma such as car accidents, falls, or sports injuries (Eli et al., 2021). According to reports, approximately 250,000 to 500,000 individuals worldwide suffer from SCI each year (James et al., 2019; Ding et al., 2022). SCI not only leads to physical dysfunctions in patients, such as loss of sensation, motor impairments, and autonomic nervous system dysfunctions, but also profoundly impacts their quality of life and socio-economic status. According to statistics, spinal cord injuries impose an annual economic burden of approximately 15.7 billion USD on society (Ma et al., 2014). Within the population of individuals with spinal cord injuries, 65% do not have the opportunity to return to employment (Trenaman et al., 2015). Epidemiological studies have shown that the incidence of SCI is higher among men, younger individuals, and in low-income countries, and is increasing with the rise in traffic accidents (Crispo et al., 2023; Shang et al., 2023; Guan et al., 2023). Spinal cord injuries impose a significant burden on society. Due to neuronal cell death and axonal transection caused by the primary injury, patients often face long-term motor and sensory dysfunctions. Currently, there are no effective treatments for SCIs, leading to a significant decline in patients’ quality of life and imposing a heavy economic and psychological burden on families and society. Globally, the incidence of SCI is rising each year. SCIs are categorized into primary and secondary injuries. Primary injuries refer to the direct damage inflicted on the spinal cord at the moment of injury, such as mechanical damage or compression. Secondary injuries are consisted by a series of pathophysiological processes that occur following the primary injury, including inflammatory responses, apoptosis, the generation of free radicals, and oxidative stress (Anjum et al., 2020). Oxidative stress is a critical factor in secondary injuries, leading to lipid peroxidation, protein denaturation, and DNA damage, further exacerbating neuronal cell death (Eli et al., 2021; Fan et al., 2018). Current treatment strategies have primarily focused on mitigating secondary injuries to minimize further damage to neural tissue and promote the recovery of neurological function. However, existing treatments still have limitations and cannot completely prevent the progression of secondary injuries. Therefore, there is an urgent need to explore new treatment strategies and drugs (Karsy and Hawryluk, 2019; Jendelova, 2018).

Prussian blue, chemically known as iron ferrocyanide, is a pigment with a distinctive blue hue. Its chemical formula is typically represented as Fe_4_[Fe(CN)_6_]_3_. It is a mixed-valence ferricyanide, composed of iron (Ⅱ) and iron (Ⅲ) ions, which combine with ferrocyanide ions in a specific ratio to form a crystalline structure (Wang et al., 2022). Prussian blue was first synthesized in the 18th century and was initially used as a pigment for its vivid and stable color, with widespread application in painting and printing industries. Due to its unique physical and chemical properties, Prussian blue has also been applied in various fields such as medicine, material science, and energy storage. Prussian blue nanotechnology is an emerging field that leverages the unique properties of Prussian blue nanoparticles, such as their high specific surface area, good biocompatibility, and adjustable physicochemical characteristics (Qin et al., 2018). These nanoparticles exhibit exceptional performance in drug delivery systems, effectively loading and releasing drugs to enhance therapeutic efficacy and bioavailability (Gautam et al., 2018). Furthermore, Prussian blue nanoparticles are also considered nanoenzymes due to their outstanding antioxidant properties, which enable them to scavenge free radicals and reduce oxidative stress. This is particularly crucial in the treatment of SCI. In the field of biomedical applications, Prussian blue nanotechnology has been explored for use in biomedical imaging, (Lu et al., 2020; Liang et al., 2023; Curdt et al., 2022), cancer treatment, (Wang et al., 2024a; Zhong et al., 2022), central nervous system disorders (Huang et al., 2022), and biological tissue engineering (Hou et al., 2022a). Its versatility makes Prussian blue a promising candidate for research in the treatment of SCI.

This article was aimed to provide a comprehensive review of the potential applications of Prussian blue nanotechnology in treating SCI and to explore possible solutions to the challenges faced by current treatment approaches. It began with an overview of the background and epidemiology of SCI, followed by a detailed discussion of the challenges and needs in SCI therapy, with a particular focus on the issue of oxidative stress in secondary injuries. The article then explored the characteristics of Prussian blue nanotechnology and its potential applications in biomedicine. It summarized specific applications of Prussian blue nanotechnology in SCI treatment, discussed the associated challenges, and outlined future research directions. Finally, the article concluded the prospects of Prussian blue nanotechnology in SCI treatment, offering new perspectives and insights for research in related fields.

## 2 Challenges and needs in SCI treatment

**FIGURE 1:**
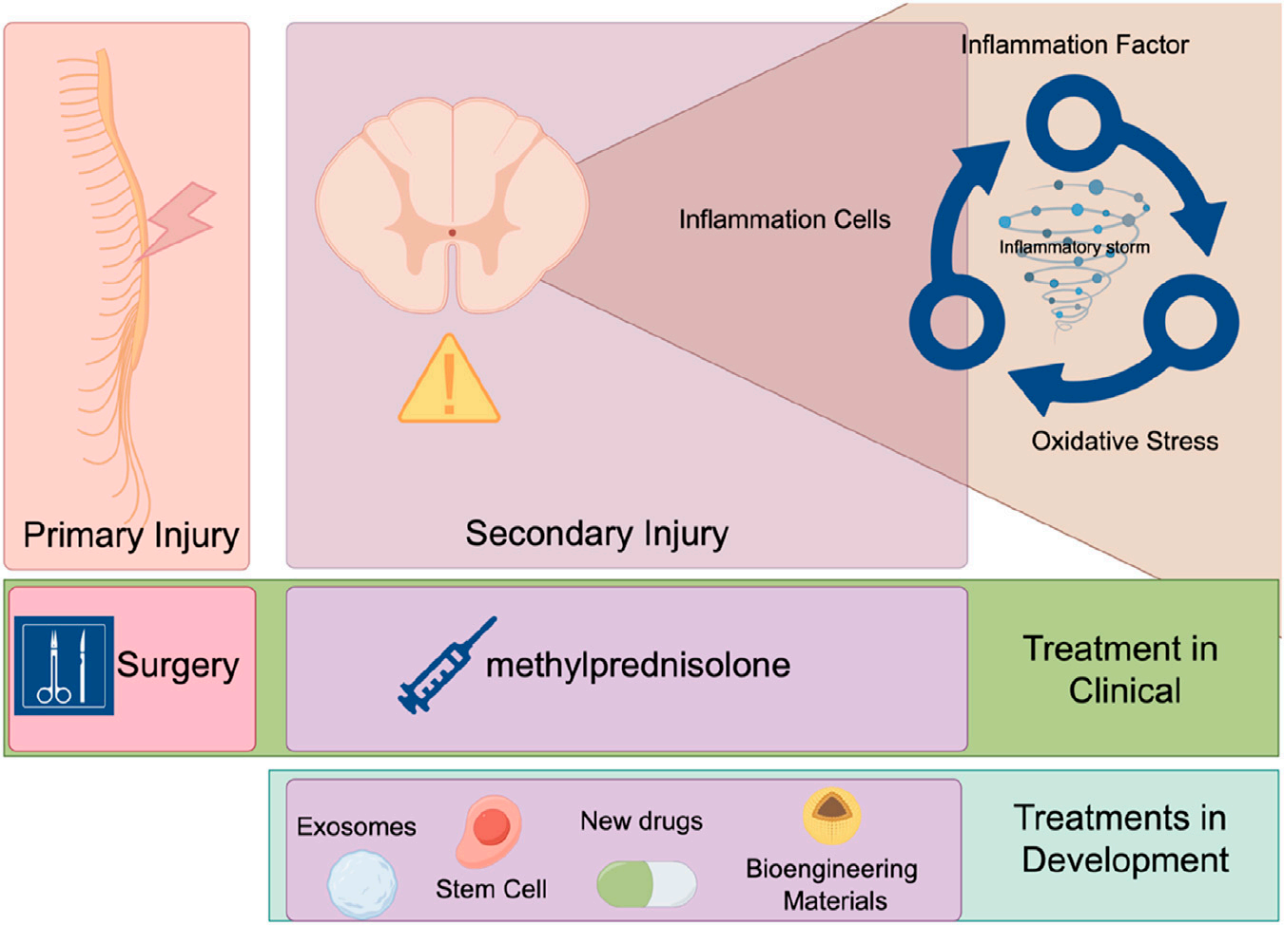
Pathology and treatment of SCI acute and subacute phases.

The pathophysiological process of SCI includes two stages: primary and secondary injuries. Primary injuries refer to the direct mechanical damage inflicted on the spinal cord at the moment of injury, typically caused by external forces such as traffic accidents, falls, or sports injuries (David et al., 2019). These forces can lead to compression, tearing, or severing of the spinal cord, resulting in immediate neuronal cell death and axonal transection. Secondary injuries involve a series of complex biological processes that rapidly begin after the primary injury and may persist for weeks to months (Li et al., 2020a; Ahuja et al., 2017). Inflammation is one of the earliest secondary injuries to occur. Following the initial damage, neutrophils and monocytes from the blood are recruited to the injury site, releasing inflammatory mediators that worsen the inflammatory response. This initiates what is known as an “inflammatory storm.” (Hellenbrand et al., 2021; Sterner and Sterner, 2022; Liu et al., 2020) Inflammatory cells release cytokines such as tumor necrosis factor-alpha (TNF-α), interleukin-1 beta (IL-1β), and interleukin-6 (IL-6), which trigger the production of additional inflammatory mediators and attract more inflammatory cells to the injury site, forming a positive feedback loop (Bretheau et al., 2022; Bigford and Garshick, 2022; O’Connor et al., 2018). These inflammatory mediators cause the relaxation of tight junctions between vascular endothelial cells, increasing vascular permeability and allowing blood components and immune cells to more easily penetrate the injury site. During inflammation, a large number of free radicals, such as superoxide anions and hydroxyl radicals, are produced. These free radicals attack cell membranes, proteins, and DNA, causing further damage (Liu et al., 2022a; Liu et al., 2023). They attack unsaturated fatty acids in cell membranes, leading to lipid peroxidation and compromising membrane integrity and function. Free radicals can also oxidize proteins, resulting in structural and functional damage that affects cellular signaling and metabolism (Cheung and Vousden, 2022). Oxidative stress can cause DNA strand breaks and base modifications, impacting gene expression and cell proliferation (Yang et al., 2019). Oxidative stress promotes the death of neurons and glial cells through various pathways, exacerbating the injury. It can also activate inflammatory cells, releasing more inflammatory mediators and creating a vicious cycle between inflammation and oxidative stress (Sies, 2015). The oxidative stress microenvironment hinders neuronal axon regeneration and the reconstruction of neural networks. Therefore, an important therapeutic strategy for addressing secondary injuries is to reduce inflammation and oxidative stress, thereby breaking the inflammatory-oxidative stress cycle (Yu et al., 2023; Zhou et al., 2020; Chio et al., 2022). Currently, in clinical practice, the primary treatments for spinal cord injuries are surgical intervention and high-dose pulse therapy with methylprednisolone. Surgical removal of compressing bone fragments is an effective method for addressing the primary injury. Surgical treatment yields better outcomes when performed early after the injury. Scholars have investigated the prognoses of patients undergoing surgery at different time intervals. A study by Jug and colleagues found that patients who underwent surgery within 8 h of injury (n = 22) had a significantly higher rate of improvement of ≥2 American Spinal Injury Association Impairment Scale (AIS) grades compared to those who had surgery between 8–24 h after injury (n = 20) (45.5% vs 10%, *p* = 0.017) (Jug et al., 2015). Fehlings recruited 313 patients with spinal cord injuries, comparing the recovery outcomes of early surgery (within 24 h) versus late surgery (beyond 24 h). The study revealed that patients who received early surgery were more likely to have an improvement of ≥2 AIS grades at the 6-month follow-up (OR 2.57, 95% CI 1.11, 5.97) (Fehlings et al., 2012). Therefore, the surgical treatment of spinal cord injuries necessitates an emphasis on “timeliness.” For the treatment of secondary injuries following spinal cord trauma, high-dose pulse therapy with methylprednisolone is the mainstay of current clinical practice. A study by Bracken and colleagues found that patients treated with methylprednisolone showed significant improvements in motor function (change scores of 16.0 vs. 11.2; *p* = 0.03), pinprick sensation (change scores of 11.4 vs. 6.6; *p* = 0.02), and touch sensation (change scores of 8.9 vs 4.3; *p* = 0.03) compared to the placebo group (Bracken et al., 1990). However, the use of corticosteroids increases the risk of bleeding, infection, and pulmonary embolism (Hejrati et al., 2023). For secondary injuries, an increasing number of safer treatment modalities are being developed. Various approaches such as stem cells (Hosseini et al., 2024), exosomes (Fan et al., 2022; Ran et al., 2023), novel pharmacological agents (Guha and Kumar, 2023; Kabu et al., 2015), and bioengineering materials (Liu et al., 2024; Liu et al., 2022b) are progressively moving from the laboratory setting into the preclinical stage (Figure 1).

## 3 Characteristics and medical applications of Prussian blue nanoparticles

Prussian Blue has garnered significant interest among researchers in the field of medical materials due to its exceptional physical properties. These properties include remarkable photothermal stability, reversible redox properties, and strong light absorption within the visible spectrum (Li et al., 2019; Cattermull et al., 2021).

The preparation methods for nanoscale Prussian blue are diverse, including chemical precipitation, hydrothermal or solvent thermal synthesis, electrochemical deposition, and template synthesis (Busquets and Estelrich, 2020). Chemical precipitation involves adding a precipitant to a solution containing iron salts and potassium ferrocyanide to form Prussian blue nanoparticles. Hydrothermal or solvent thermal synthesis uses high temperatures and pressures in water or organic solvents to produce nanoparticles, allowing for better control over particle size and morphology. Electrochemical deposition applies voltage to an electrode surface, where iron ions and ferrocyanide ions react directly on the electrode to form a Prussian blue film. Template synthesis adopts templates, such as porous membranes, to guide particle growth, resulting in Prussian blue nanoparticles with specific shapes and sizes (Busquets and Estelrich, 2020). The biological characteristics of nanoscale Prussian blue are crucial in determining its suitability as a biomaterial for biomedical applications. These characteristics include its antioxidant properties, photothermal performance, biocompatibility, and imaging capabilities (Lu et al., 2023; Komkova and Karyakin, 2022; Du and Hou, 2023).

### 3.1 Antioxidant and anti-inflammatory properties

Nanoscale Prussian blue exhibits excellent antioxidant properties, allowing it to effectively scavenge free radicals such as superoxide anions and hydroxyl radicals, thereby reducing oxidative stress-induced damage to cells. Prussian blue nanoparticles can inhibit lipid peroxidation, protecting cell membranes from oxidative damage (Chen et al., 2023a). They also safeguard proteins and DNA from oxidative damage, thereby maintaining normal cell function. Furthermore, these nanoparticles can inhibit the infiltration and activation of inflammatory cells, reducing both their number and activity (Zhang et al., 2021). These characteristics make Prussian blue nanoparticles potentially valuable for treating SCI and other diseases associated with oxidative stress. For example, in the case of ischemic stroke, local injection of hollow Prussian blue nanoparticles can reduce oxidative stress, protect damaged neural tissue, and promote the recovery of neurological function (Zhang et al., 2019; Liu et al., 2021; Huang et al., 2018). In liver injury, Prussian blue nanoenzymes can alleviate oxidative stress, regulate inflammation, and protect liver cells through Nrf2-related signaling pathways (Bai et al., 2021a).

### 3.2 Photothermal properties

The photothermal properties of Prussian blue arise from its unique crystal structure and electronic characteristics, which enable it to effectively absorb visible and near-infrared light. The charge transfer transitions between the iron (Ⅱ) and iron (Ⅲ) ions in Prussian blue allow it to absorb specific wavelengths of light energy, making it highly effective in photothermal conversion. The light absorption range of Prussian blue typically spans from 400 to 900 nm, covering most of the solar spectrum. When Prussian blue absorbs light energy, it converts it into heat energy through a process known as photothermal conversion. Prussian blue demonstrates relatively high photothermal conversion efficiency, meaning it can generate a significant amount of heat from a relatively small amount of light energy. This efficient photothermal conversion capability makes Prussian blue valuable for applications such as photothermal therapy and photothermal energy conversion. For instance, Prussian blue can function as a photothermal agent in photothermal therapy. Under near-infrared light irradiation, Prussian blue nanoparticles can locally generate heat to kill cancer cells or destroy tumor vessels (Tang et al., 2023; Hua et al., 2021; Sun et al., 2021).

### 3.3 Biocompatibility

Biocompatibility refers to a material’s ability to interact with biological tissues without causing harmful physiological reactions over prolonged periods. The biocompatibility of Prussian blue nanoparticles is primarily evident in their low toxicity, biodegradability, non-biological activity, and potential for surface modification. Prussian blue nanoparticles exhibit good biocompatibility within the body, showing no significant toxicity (Wang et al., 2020). They remain stable in the body’s environment and, at appropriate concentrations, do not trigger immune reactions or cytotoxicity (Wang et al., 2022). These nanoparticles can gradually degrade under specific conditions, releasing any bioactive substances they carry while minimizing potential long-term toxicity. This degradation process can be controlled by altering the composition, size, and surface properties of the nanoparticles. Prussian blue nanoparticles do not possess biological activity that would interfere with normal physiological functions when used as drug delivery carriers or imaging contrast agents (Dumani et al., 2020). Their biocompatibility can be further enhanced through surface modification, such as conjugation with biocompatible polymers or biomolecules, which helps reduce non-specific interactions with biological systems. Nanoscale Prussian blue demonstrates good biocompatibility, allowing it to remain stable within the body’s environment without causing significant immune reactions or toxicity. This is particularly important for long-term implantable drug delivery systems (Cho et al., 2023). Due to its exceptional biological properties, Prussian blue is increasingly utilized in medical research.

### 3.4 Drug delivery systems

Prussian blue nanoparticles, with their unique cubic structure and adjustable surface properties, offer several advantages as drug delivery carriers, including blood stability, biocompatibility, biodegradability, low cytotoxicity, cost-effectiveness, ease of preparation, tunable morphology, and size control. These properties make Prussian blue nanoparticles suitable for meeting various drug delivery requirements, such as high drug loading efficiency and targeting specificity (Chen et al., 2023b; Da et al., 2022). In research, Prussian blue nanoparticles are frequently used to load drugs or target specific molecules (Gao et al., 2020). For example, Zhang et al. adopted Prussian blue nanoparticles to deliver two antitumor drugs, daunorubicin and cytarabine, achieving synergistic effects through combined chemotherapy and photothermal therapy (Bai et al., 2021b). Similarly, Wang et al. enhanced the therapeutic effects of drugs by combining Prussian blue with nanoparticles coated with tumor cell membranes, which improved targeted delivery and reduced side effects (Ma et al., 2024).

### 3.5 Contrast agents

Magnetic Resonance Imaging (MRI) is a widely used imaging technique in medicine. Researchers have discovered that Prussian blue can serve as a contrast agent for MRI (Lee et al., 2015; Zhu et al., 2020). Prussian blue nanoparticles enhance image contrast and clarity in medical imaging. Although its contrast in T1-weighted images is lower compared to commercial contrast agents, Prussian blue remains of significant interest, particularly because it offers integrated diagnostic and therapeutic effects (Peng et al., 2018). In addition to MRI, Prussian blue can also be used as an ultrasound contrast agent (Tian et al., 2017; Zhang et al., 2016).

### 3.6 Biological sensor

Prussian blue nanoparticles are increasingly being recognized for their applications in the field of biosensors. They can detect specific molecules within the body, such as glucose, DNA, and proteins (Ben Hassine et al., 2023).This capability provides a new tool for the early diagnosis of diseases like diabetes (Tong et al., 2023) and tumors (Gao et al., 2020).

**FIGURE 2:**
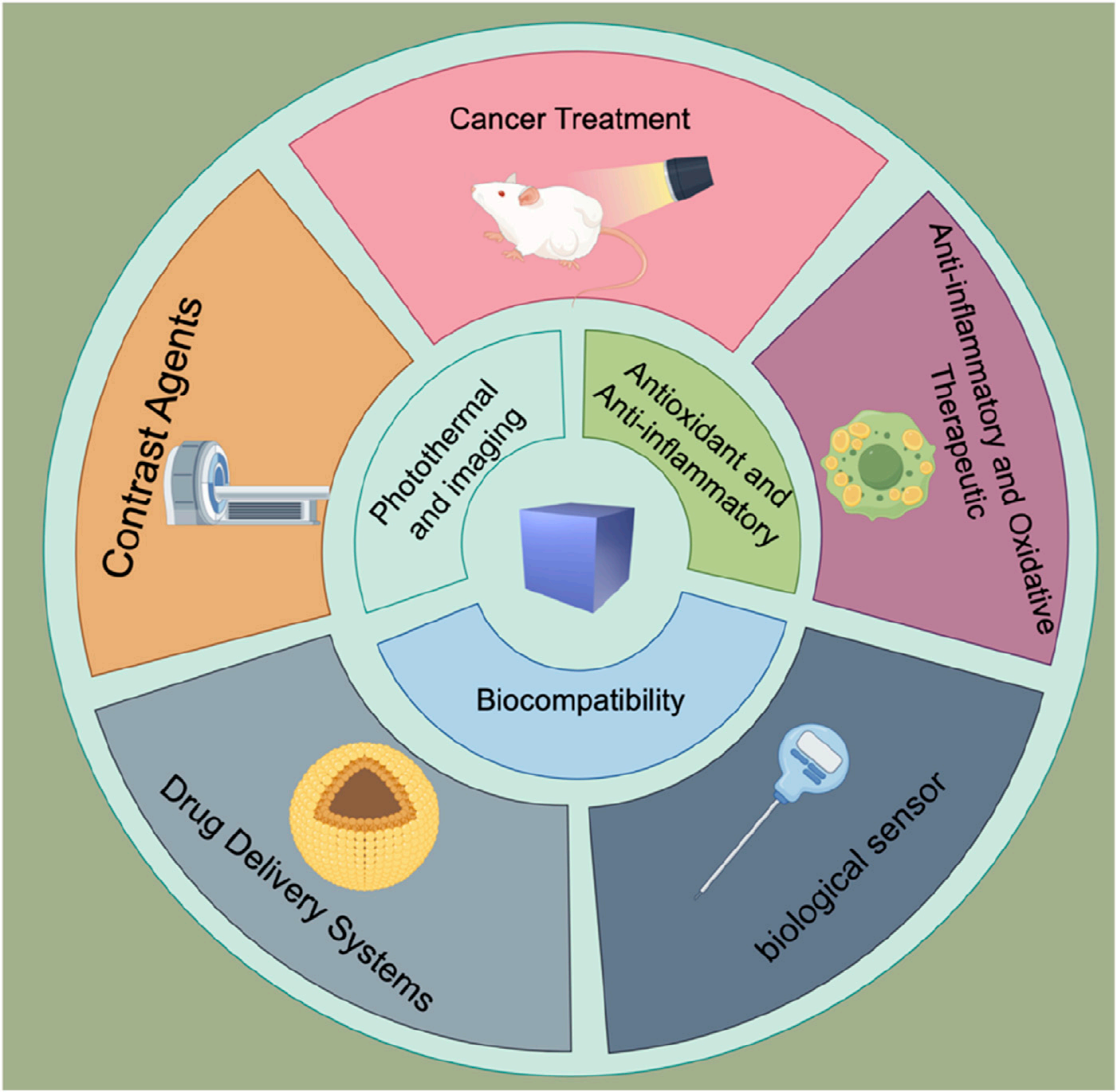
Characteristics and medical applications of Prussian blue nanoparticles.

The clinical applications of Prussian blue nanoparticles highlight their significant potential in the future development of medicine, particularly in personalized treatment and precision medicine. As research continues to advance, the use of Prussian blue nanotechnology in clinical applications is expected to become more widespread (Figure 2).

## 4 Application of Prussian blue nanotechnology in the treatment of SCI

Functional nanomaterials are rapidly advancing in the field of SCI treatment research, demonstrating broad application prospects. These nanomaterials can be broadly categorized into organic and inorganic types based on their chemical composition. Among organic nanomaterials, polymer nanoparticles, liposomes, and exosomes are at the forefront of research. Polymer nanoparticles are favored for their biodegradability and controlled drug release capabilities; however, challenges such as immunogenicity and drug leakage need to be addressed (Gao et al., 2017; Lu et al., 2018). Liposomes are renowned for their excellent biocompatibility and high drug-loading capacity, but their limited stability and the need for improved targeting are constraints on their application (An et al., 2022; Wang et al., 2018a). Exosomes, as natural nanovesicles, exhibit superior membrane fusion abilities and low immunogenicity, yet their purification and large-scale production present technical hurdles (Fan et al., 2022; Ran et al., 2023; Yu et al., 2024).

Common representatives of inorganic nanoparticles include gold nanoparticles, iron oxide nanoparticles, and silica nanoparticles. Gold nanoparticles excel in photothermal therapy and imaging due to their unique optical properties, but their biodegradability and long-term toxicity require further investigation (Ozcicek et al., 2024; Ko et al., 2021). Iron oxide nanoparticles stand out as contrast agents in magnetic resonance imaging (Zhang et al., 2024a; Pal et al., 2013). Silica nanoparticles are widely used for their high stability and ease of surface functionalization, yet their biodegradability and long-term biocompatibility remain concerns (Wang et al., 2018b). Prussian blue, an inorganic nanoparticle, has particularly notable advantages in SCI treatment. It not only possesses potent antioxidant capabilities, effectively mitigating oxidative stress damage, but also exhibits excellent biocompatibility, reducing potential immune responses (Yuan et al., 2024). Moreover, the multifunctionality of Prussian blue enables its utility in both imaging and therapeutic applications (Wang et al., 2024b; Sweeney et al., 2024), which is particularly important in SCI treatment. Consequently, Prussian blue not only shows tremendous potential in the realm of SCI treatment but also offers new insights and directions for future clinical applications. The following is a summary of the current research on Prussian blue in the context of spinal cord injury.

### 4.1 Stem cell tracking

Stem cell therapy offers promising potential for the treatment of SCI, but it has not yet achieved effective clinical application. One barrier to its clinical translation is the challenge of monitoring the functional performance of stem cells (Donnelly et al., 2012; Syková and Jendelová, 2006). MRI is a preferred method for monitoring Prussian blue nanoparticles within spinal cord tissue (Lamanna et al., 2017). Research has shown that when human umbilical cord mesenchymal stem cells labeled with Prussian blue are injected into the spinal cords of rats, MRI can detect the labeled cells for up to 8 weeks. This provides evidence of the proliferation and migration of stem cells within the spinal cord (Hu et al., 2012). However, MRI is not always convenient for practical operations. To address this, ultrasound is also used to study Prussian blue-assisted stem cell therapy. Kim et al. found that mesenchymal stem cells labeled with Prussian blue could be detected by optical signals for up to 14 days after injection (Kim et al., 2017). Emelianov et al. discovered that ultrasound can be used to guide the injection needle in real-time and monitor the trajectory of neural stem cells. Furthermore, the stem cell tracks were found to be consistent across both ultrasound and MRI detection methods. Thus, Prussian blue labeling is a promising method for real-time monitoring of stem cell therapy in SCI (Kubelick and Emelianov, 2020a). Additionally, Prussian blue enables multimodal monitoring that combines ultrasound, optical, and MRI techniques (Kubelick and Emelianov, 2020b; Kubelick and Emelianov, 2020c).

### 4.2 Detection and treatment of iron ions

Prussian blue, traditionally used as a dye, also has applications in the histological staining of SCI tissues. Ferroptosis, a novel form of programmed cell death, has been identified as a significant pathological process in secondary injuries following SCI (Shi et al., 2021; Li et al., 2023a; Hu et al., 2022). This type of cell death is associated with the accumulation of iron in the neurons surrounding the injury site, which can trigger ferroptosis (Li et al., 2023b; Li and Jia, 2023). Prussian blue nanoparticles are valuable for detecting iron ions because they chemically react with iron ions to form a complex that can be detected through changes in color, spectral properties, or electrochemical characteristics. For instance, Prussian blue nanoparticles can serve as a biosensor for iron ions, useful for detecting their concentration in the human body or monitoring iron pollution in the environment. Thus, Prussian blue can be used to stain SCI tissues to indicate the accumulation of iron ions or the occurrence of ferroptosis (Feng et al., 2021; Zhang et al., 2024b). Moreover, Prussian blue can detect iron-labeled stem cells, providing insights into their location and status within the body (Zhang et al., 2013; Lei et al., 2009; Zhang et al., 2015). It can also be used to assess whether iron-labeled drugs reach the injured area (Liu et al., 2010). Targeting ferroptosis is a prominent area of research for SCI treatment. Various ferroptosis inhibitors (Guo et al., 2023; Kang et al., 2023; Shen et al., 2024) have been found to improve functional recovery after SCI. The Nrf2/GPX4 pathway (Ge et al., 2021), P53-ALOX15 pathway (Li et al., 2023b), and the STING signaling pathway (Hu et al., 2022) were found to be therapeutic targets for SCI. Given Prussian blue’s ability to bind iron ions, it may have potential as an inhibitor of ferroptosis. Studies have shown that Prussian blue can alleviate ferroptosis in inflammatory bowel disease. Zhu et al. (2023), but its effects on ferroptosis in SCI have not yet been reported in the literature. This presents a promising avenue for future research.

### 4.3 Nanoparticles modified with Prussian blue improved the microenvironment of the injured area

Prussian blue exhibits strong antioxidant properties and high stability. It is an FDA-approved reductant for treating thallium or cesium poisoning. Given the high levels of reactive oxygen species (ROS) present in the SCI microenvironment, Prussian blue is a promising candidate for therapeutic use in SCI. Prussian blue is renowned for its unique structure that effectively scavenges reactive oxygen species (ROS). The nanoparticles of Prussian blue contain iron (II) and iron (III) ions, which are arranged in its lattice structure to create an environment conducive to electron transfer. When Prussian blue nanozymes come into contact with ROS, the iron ions within the nanozymes undergo oxidation, thereby neutralizing the ROS. This process mimics the activity of catalase (Xu et al., 2022). Research has demonstrated that Prussian blue can significantly reduce ROS accumulation in the injured areas of the central nervous system (Zhang et al., 2019). In addition to alleviating oxidative stress, Prussian blue also exhibits anti-inflammatory properties. The anti-inflammatory characteristics of Prussian blue are multifaceted: on one hand, its ability to scavenge ROS mitigates the initiation of subsequent inflammatory responses. On the other hand, Prussian blue can modulate immune cells, promoting the polarization of macrophages towards the M2 phenotype, which in turn reduces the release of inflammatory cytokines (Da et al., 2022; Hou et al., 2022b).

**FIGURE 3:**
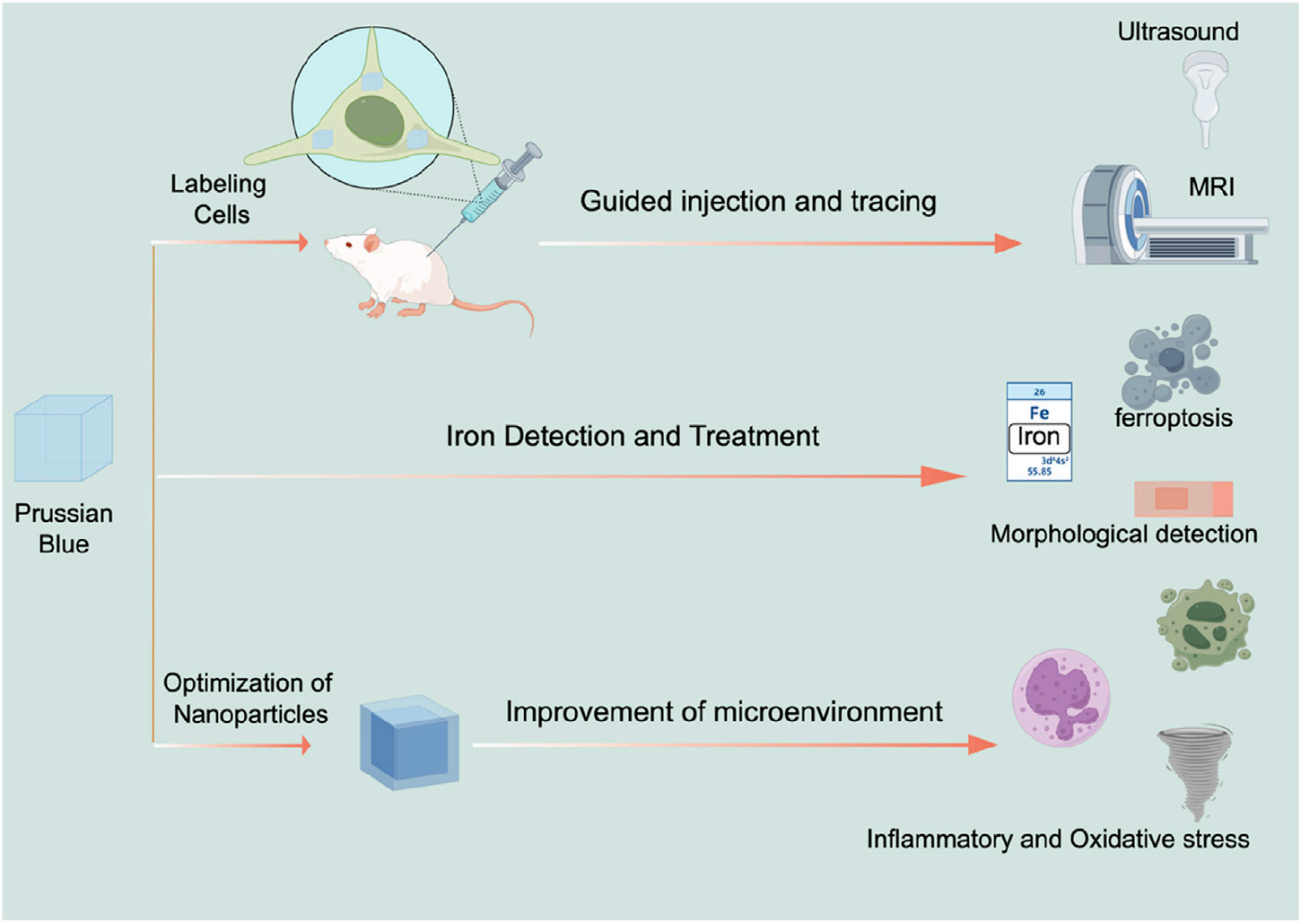
The application of Prussian blue in SCI treatment.

Moreover, combining Prussian blue with other nanomaterials can further enhance its antioxidant properties. Gao et al. investigated the combination of Prussian blue with nanoscale zirconium for SCI treatment (Gao et al., 2024). Their findings suggest that Prussian blue-zirconium nanoparticles not only improved the oxidative stress microenvironment but also alleviated functional disorders in neurons and macrophages, potentially due to zirconium’s role in regulating zinc ion deposition (Gao et al., 2024). Prussian blue can also be combined with traditional drugs to improve their therapeutic efficacy for SCI. Gao et al. developed a composite nanoparticle by combining Prussian blue with schisandrin, encapsulated within a zeolitic imidazolate framework-8 (ZIF-8) nanoparticle platform. This composite not only inhibited ROS but also induced macrophages to polarize towards the M2 phenotype (Lin et al., 2024), highlighting the potential of Prussian blue modifications in creating multifaceted treatment approaches. Following SCI, the expression of matrix metalloproteinases (MMPs) increases in the injured microenvironment (Yao et al., 2018). Activating cell-penetrating peptides targeting MMPs can be used to specifically address the injury area (Shen et al., 2021). Shen et al. combined activating cell-penetrating peptides with Prussian blue nanoparticles, resulting in a composite nanoparticle that improves the oxidative stress microenvironment and exhibits good targeting properties for the injury site (Shen et al., 2023). Recent research has also focused on using extracellular vesicles (EVs) to encapsulate nanoparticles (Mondal et al., 2023; Dad et al., 2021; Stine et al., 2020). EVs can target specific cell types, facilitating targeted treatment of key cells involved in SCI. Zhang et al. used macrophage-derived EVs to encapsulate manganese-iron Prussian blue analogues (Bai et al., 2024). Their study found that these exosome-encapsulated Prussian blue nanoparticles effectively targeted microglia, significantly promoting microglial aggregation and improving oxidative stress and inflammation in microglia (Bai et al., 2024). This suggests that exosome-encapsulated Prussian blue is a viable and promising treatment approach (Figure 3) (Table 1).

**TABLE 1:** Application of Prussian blue in spinal cord injury treatment.

Application areas	Concentration of prussian blue	Application methods	Effect	References
Cell tracing	53 ug/mL	Local injection	Stem cells labeled with Prussian blue can be monitored under ultrasound and MRI	Kubelick and Emelianov (2020a), Kubelick and Emelianov (2020b), Kubelick and Emelianov (2020c)
Microenvironment improvement	Not given	Intraperitoneal injection	Prussian blue and zirconium can synergistically improve inflammation, oxidative stress and apoptosis in spinal cord injury	Gao et al. (2024)
Microenvironment improvement	10 μg/mL	Intravenous injection	Prussian blue and rapamycin-combined nanoparticles inhibit inflammation and apoptosis by inhibiting the MAPK/AKT signaling pathway	Shen et al. (2023)
Detecting iron deposition	—	Morphological staining	Prussian blue is a good indicator of iron deposition in tissues or iron-labeled cells	Hu et al. (2012), Zhang et al. (2024b), Zhang et al. (2013), Blomster et al. (2013)

## 5 Summary and outlook

Prussian blue nanotechnology has demonstrated significant potential in the treatment of SCI, largely due to its potent anti-inflammatory and antioxidant properties, which make it an attractive option for therapeutic intervention. However, several challenges must be addressed before it can be widely applied in clinical settings. The pathological mechanism of spinal cord injury is complex, and a single antioxidant treatment is insufficient to achieve the desired effect (Zhang et al., 2024c). Combining molecules with additional functions, such as promoting angiogenesis (Miao et al., 2024), facilitating axon growth (Fan et al., 2022), and others, may lead to improved therapeutic outcomes. Currently, intravenous injection has been the most common method for administering Prussian blue nanoparticles in clinical practice. However, due to the lack of specific targeting capabilities, these nanoparticles may distribute unevenly throughout the body, which can diminish their therapeutic effectiveness. Nanoparticles predominantly accumulate in the liver. This accumulation effect not only diminishes the efficacy of nanoparticles at the target site but also increases the burden on the liver (Li et al., 2020b). To enhance the targeted delivery of Prussian blue nanoparticles to the SCI site, improving their targeting specificity is crucial. It can be achieved by modifying their surface properties or by combining them with other targeting molecules. For instance, coating nanoparticles with molecules such as C-C chemokine receptor type 2 and trans-activator of transcription, which have the ability to target spinal cord injury sites, can enhance the accumulation of nanoparticles in the region of spinal cord injury (Gu et al., 2024; Li et al., 2021). Local injection presents an alternative approach that could improve the efficacy of Prussian blue nanoparticles in SCI treatment. By administering the nanoparticles directly to the injury site, the drug can be more concentrated, potentially enhancing its therapeutic effect. Additionally, modifying the local sustained release of Prussian blue nanoparticles could be a promising research direction. For instance, combining these nanoparticles with hydrogels or other carriers could facilitate a prolonged release of the drug at the injury site, thereby extending its therapeutic duration. The microenvironment at the site of spinal cord injury is rife with ROS, which severely impede the survival and efficacy of stem cells in the injured area (Liu et al., 2023). Prussian blue nanoparticles possess potent antioxidant properties and can significantly improve the microenvironment of spinal cord injury. Combining Prussian blue with stem cell therapies may further enhance the effectiveness of stem cell treatments.

In summary, Prussian blue nanotechnology holds significant promise for SCI treatment. However, the application of Prussian blue in spinal cord injury is still in the preliminary exploratory stage. In this review, we proposed several potential research ideas for the application of Prussian blue in spinal cord injury. Further research is needed to overcome existing challenges and optimize its clinical application. The potential for this technology in personalized and precision medicine is substantial, and as research advances, its application in clinical settings is expected to become more widespread.
